# Dental Caries

**Published:** 1879-10

**Authors:** C. N. Pierce

**Affiliations:** Philadelphia


### DENTAL CARIES.

BY C. N. PIERCE, D. D. S., PHILADELPHIA.

*Read before the Pennsylvania State Dental Society.*

Dr. Pierce read the following paper upon the causes of caries, and therein discussed the views that have been advanced by different authors. In it he reviewed the different theories which have been advanced by various authors and members of the profession: such as chemical, vital, chemico-vital, parasitic, and the electric. He thought the varying qualities of tooth structure, and the diverse systemic conditions, rendered the adoption of any positive views respecting either the cause or treatment of caries very difficult. Our education has been too limited, and our statistics too incomplete for any satisfactory classification or arrangement. He thought the pathological process, which results in the greater or less destruction of tooth tissue, and which is recognized under the name of dental caries, belongs largely to civilized life; its growing increase, and now almost universal prevalence being a concomitant of modern civilization. It was a question well worth considering, whether many of the various conditions now recognized as being present during the progress of Dental caries, and supposod to facilitate its advance, be not like the disease itself, the result of some modified life force, and occurring simultaneously as an effect of the same cause. We take for instance what is recognized as the parasitic cause of caries. Some believe it to be due to, or greatly facilitated by, a putrefaction which is, or was supposed to be, caused by a mass of minute infusorial animalcules which live in the mouth, and to which they have given the name "Denticola." Others think it due to putrefaction, caused by a rapid proliferation of a vegetable parasite called "Protococcus Dentalis," or to another parasite which burrows into the tissues of the teeth, known as "Leptothrix Buccalis." Earnest, honest, and apparently accurate descriptions have been given of the histologic changes of the enamel and dentine, as the destruction of these tissues gradually progressed under the supposed influence of these parasites.

The authors and advocates of the parasitic theory seem to have overlooked the fact, that it had been, and indeed still is, an open question, whether these microscopic beings, vegetable or animal, are more than mere followers of decay, "messmates," lying always in wait to consume the tissues of animals and plants, following devitalization and disorganization, never preceding. Electrolysis, the still more modern theory, and upon which is based the "New Departure," is not less enthusiastically presented by its advocates, but it may yet be found to be the result of other than local conditions. This subtle enemy, which some of our friends have so satisfactorily to themselves, demonstrated to be the destroying agent, is supposed to be the result of an abnormal condition of the tissues of the teeth. To use the language of Mr. R. Bridgman, "The pulp-vessels should be charged with negative electricity; but in a pathological state, the surface of the dentine, as well as the roots, would be charged with negative electricity." Now of the existence of these positive and negative conditions, and of the currents resulting therefrom, we know but little, nor have we as yet, to my knowledge, any method of accurately measuring or testing their presence or quality. It is assumed with a degree of authority, that all chemical action involves electric disturbance, and also that acid fluids act chemically on all tissues in which the salts of lime largely preponderate, as in tooth structure. It is also asserted, and with so much earnestness and decision, that it is fair to assume that this is the corner-stone of the "New Departure," that this electro-chemical action is greatly facilitated by the presence of a gold filling in the tooth; that its disturbing influence is due largely to its capability of conducting thermal changes to the surrounding tissue, and also that fillings of other materials are more and more in harmony with the normal electrical condition of the tooth, in proportion as they possess a poorer conducting power. Now, of the theories which have been advanced, it is a natural query, whether this one of electrolysis is, any more than the others, capable of satisfactorily explaining conditions which time and again seem to baffle all our best efforts in tooth saving. To assert dogmatically, that gold is the best material with which to save all teeth, would be a statement which, in my estimation, would be very wide of the truth. That gold has been effectually used, and can be, and is so used in full two-thirds of all the teeth which need filling, is certainly a statement in correspondence with the judgment of the majority of educated dentists. That the remaining third can be as well saved, and probably better, with some other material than gold, will, I think, be admitted by the majority of our practitioners. But I am not prepared to state why gutta-percha, oxychloride of zinc, amalgam, or tin, in some form, has a more benign influence upon a certain kind of tooth structure than gold, other than the fact of their increased adaptability and decreased conductibility. The fact that thousands of gold fillings can be found in teeth, where they have been doing good service for twenty years and upwards, is pretty conclusive evidence that these teeth at least have not had their electrical condition disturbed sufficiently to produce any destruction of tooth-tissue.

Dr. Pierce also exhibited three drawings, which will be found with their extended explanations in the *Cosmos* for September, 1878, illustrating the movements of the lower jaw, and endeavored in the following remarks, to show how the movements of the mandible had modified the shape and structure of the teeth.

He said the articulation of the mandible with the skull presents three well marked types, viz: That observed, 1st. with a more or less cylindroidal mandibular condyle. 2d That with a transversely-expanded and superiorly plane condyle. 3d. That with a more or less nearly globular condyle, which, in forms, with much reciprocating movement, becomes elongated antero-posteriorly, narrowed transversely, and fits, in some instances, into a well-marked, longitudinally disposed glenoid cavity. In those forms in which there is much lateral motion, the rami of the mandible are much more closely approximated than the molar series of the opposite sides above, while in those forms without the excursive lateral movement, there is little or no such difference in the interval between the molars of the upper and lower series respectively.

It is, therefore, apparent, that the relations supposed to exist between lower jaw movement and tooth-forms, must be extended so as to embrace not only changes in the form and strength of the teeth, but also changes in the relations of the mandible, and the form of their condyles and the glenoid cavities. The whole of these changes have been dependent upon the mode of use of the parts and the force exerted.

It is further to be observed, that those animals which have the most powerful masticating apparatus, invariably have the most deeply implanted teeth, and the most effective arrangement for the replacement of tooth-structure when lost by wear. It is also apparent, that as the excursive movements have increased in complexity, there has been an increase in the complexity of the enamel foldings, ridges and crests, from the fact that the foldings, ridges and crests, have apparently been modified in conformity to the ways in which the force used in mastication was exerted. It is therefore concluded that the various modes of crest and tubercular modification are related as effects to the diverse modes of mandibular movement.

These views, based as they are upon the diversity of development found throughout the mammalia, must have some bearing upon the rapid degeneration of teeth in the human species, and point very conclusively to a want of a proper use of the teeth in comminuting harder food, as a prolific cause, of some of the almost universal prevalence of dental caries and other dental deformities.

The use of solid foods would, doubtless, be a constant active stimulant, tending to produce a healthier and stronger development, while the long established and increasing use of soft foods must have an unmistakable tendency to cause the teeth to become diseased; and if there be truth in the doctrine of hereditary transmission, will in time render the human family more or less edentulous.—*Transactions Pennsylvauia Dental Society.*

